# Transcriptomic analysis of caecal tissue in inbred chicken lines that exhibit heritable differences in resistance to *Campylobacter jejuni*

**DOI:** 10.1186/s12864-021-07748-2

**Published:** 2021-06-04

**Authors:** Kay M. Russell, Jacqueline Smith, Abi Bremner, Cosmin Chintoan-Uta, Lonneke Vervelde, Androniki Psifidi, Mark P. Stevens

**Affiliations:** 1 The Roslin Institute and Royal (Dick) School of Veterinary Studies, University of Edinburgh, Easter Bush, Midlothian, EH25 9RG UK; 2 The Royal Veterinary College, Hawkshead Lane, Hatfield, Hertfordshire, AL9 7TA UK

**Keywords:** *Campylobacter jejuni*, Chicken, Resistance, Susceptibility, Transcriptome, Gene expression, Inbred, RNA-Seq

## Abstract

**Background:**

*Campylobacter jejuni* is the leading cause of bacterial gastroenteritis in humans and the handling or consumption of contaminated poultry meat is a key source of infection. Selective breeding of poultry that exhibit elevated resistance to *Campylobacter* is an attractive control strategy. Here we studied the global transcriptional response of inbred chicken lines that differ in resistance to *C. jejuni* colonisation at a key site of bacterial persistence.

**Results:**

Three-week-old chickens of line 6_1_ and N were inoculated orally with *C. jejuni* strain M1 and caecal contents and tonsils were sampled at 1 and 5 days post-infection. Caecal colonisation was significantly lower in line 6_1_ compared to line N at 1 day post-infection, but not 5 days post-infection. RNA-Seq analysis of caecal tonsils of both lines revealed a limited response to *C. jejuni* infection compared to age-matched uninfected controls. In line N at days 1 and 5 post-infection, just 8 and 3 differentially expressed genes (DEGs) were detected (fold-change > 2 and false-discovery rate of < 0.05) relative to uninfected controls, respectively. In the relatively resistant line 6_1_, a broader response to *C. jejuni* was observed, with 69 DEGs relating to immune regulation, cell signalling and metabolism at 1 day post-infection. However, by day 5 post-infection, no DEGs were detected. By far, the greatest number of DEGs were between uninfected birds of the two lines implying that differential resistance to *C. jejuni* is intrinsic. Of these genes, several Major Histocompatibility Complex class I-related genes (*MHCIA1*, *MHCBL2* and *MHCIY*) and antimicrobial peptides (*MUC2*, *AvBD10* and *GZMA*) were expressed to a greater extent in line N. Two genes within quantitative trait loci associated with *C. jejuni* colonisation were also more highly expressed in line N (*ASIC4* and *BZFP2*). Quantitative reverse-transcriptase PCR analysis of a subset of transcripts confirmed the RNA-Seq results.

**Conclusions:**

Our data indicate a limited transcriptional response in the caecal tonsils of inbred chickens to intestinal colonisation by *Campylobacter* but identify a large number of differentially transcribed genes between lines 6_1_ and N that may underlie variation in heritable resistance to *C. jejuni*.

**Supplementary Information:**

The online version contains supplementary material available at 10.1186/s12864-021-07748-2.

## Background

*Campylobacter* is estimated to have caused 95 million cases of acute gastroenteritis in humans in 2010, with the loss of 21,000 lives and 2.1 million disability-adjusted life years [1]. In the United Kingdom alone, 63,946 laboratory-confirmed cases of human campylobacteriosis were recorded in 2017 [2] and a further 9.3 cases were predicted to be unreported for every one captured by national surveillance [3]. Such infections have been estimated to cost the UK economy approximately £50 million per annum through lost productivity and healthcare costs [4]. *Campylobacter* infections in humans often involve watery diarrhoea, abdominal cramps and nausea but generally resolve within a week [5]. However, infections can relapse and severe sequelae exist, including inflammatory neuropathies such as the Guillain-Barré syndrome [5].

Source attribution studies unequivocally implicate the handling or consumption of contaminated poultry meat as a key risk factor for human campylobacteriosis, with up to 80% of cases thought to be attributable to the avian reservoir [6, 7]. The caeca are a key site of persistence of *Campylobacter* in poultry, where numbers of *C. jejuni* can reach as high as 10^10^ colony forming units (CFU)/g of contents in the absence of overt pathology. Given such levels, contamination of carcasses with numbers of *C. jejuni* predicted to be adequate for human infection is challenging to prevent during the slaughter process [8]. A recent survey in the United Kingdom found that 54% of fresh retail chicken was contaminated with *Campylobacter* [9]. Birds generally become colonized with *C. jejuni* from their environment, and across Europe the prevalence of *C. jejuni* positive flocks ranges from 18 to 90%, with seasonal variation and the highest levels occurring in the summer months [10]. Control of *Campylobacter* in poultry primarily relies on stringent on-farm biosecurity measures and carcass treatments. No commercial vaccines exist and it is likely that a multifactorial approach will be required.

It has been estimated that a 2 log _10_ reduction in the level of poultry carcass contamination by *C. jejuni* could lower the incidence of human cases due to this source by 12 to 30-fold [11]. However, more modest estimates suggest a 3 log_10_ reduction in caecal colonisation would reduce human cases by 58%, although with a high degree of uncertainty [12]. One option to achieve this is to improve the intrinsic resistance of chickens to *Campylobacter* colonisation. Differences exist in the levels of colonisation across and within commercial broiler lines [13, 14] and these have been associated with variation in the transcriptome of the caeca [14–16] and spleen [17]. Genome-wide association studies in a commercial broiler population have indicated that resistance to caecal *C. jejuni* colonisation is under moderate genetic control [14]. However, heritable differences in resistance have been associated with quantitative trait loci (QTL) [14, 18], and the transcription of genes related to immunity [14, 19].

White Leghorn-derived inbred chicken lines 6_1_ and N have been reported to be relatively resistant and susceptible to colonisation by several *C. jejuni* strains, respectively [20, 21], with F1 progeny of a cross exhibiting intermediate phenotypes [20]. Genome-wide association studies using backcross [(6_1_ x N) x N] and ninth generation advanced intercross (6_1_ x N) populations have identified QTLs associated with resistance to caecal colonisation by *C. jejuni* in these lines [21]. In this study, two candidate genes were identified in the QTL regions, *ASIC4*, located on chromosome 7, and *BZFP2* located on chromosome 16, indicating a potential association with the Major Histocompatibility Complex (MHC) locus also present on chromosome 16. Immune-related genes such as *IL6*, *CXCLi2* and *CCLi2* [22] and immune-related pathways including lymphocyte activation, cytokine signalling and Ig production [15, 17, 19] have also been proposed to contribute to the differential resistance of chicken lines. Irrespective of the association of genes or expression patterns with heritable resistance, a need exists to better understand how birds respond to *C. jejuni* during infection, where previous studies have suggested a pro-inflammatory response that is limited in magnitude and timing [23–26], but which may also differ between commercial broiler lines [27].

Line 6_1_ and N chickens not only differ in resistance to *Campylobacter*, but to gut colonisation by *Salmonella enterica* serovar Typhimurium and genetic associations have been mapped using a backcross [28]. We recently demonstrated that limited differences exist between lines 6_1_ and N in their caecal microbiota and reciprocal transplants of caecal microbiota did not alter their resistance to *C. jejuni* colonisation, suggesting a role for host factors [29]. Here, we used RNA-Seq to investigate the caecal transcriptome of line 6_1_ and N chickens, both in uninfected birds to identify differences between the lines that may underlie differential resistance to pathogens and following experimental challenge with *C. jejuni*.

## Results

### Challenge of line 6_1_ and line N birds with *C. jejuni* M1 confirms differential resistance early after inoculation

**Fig. 1:**
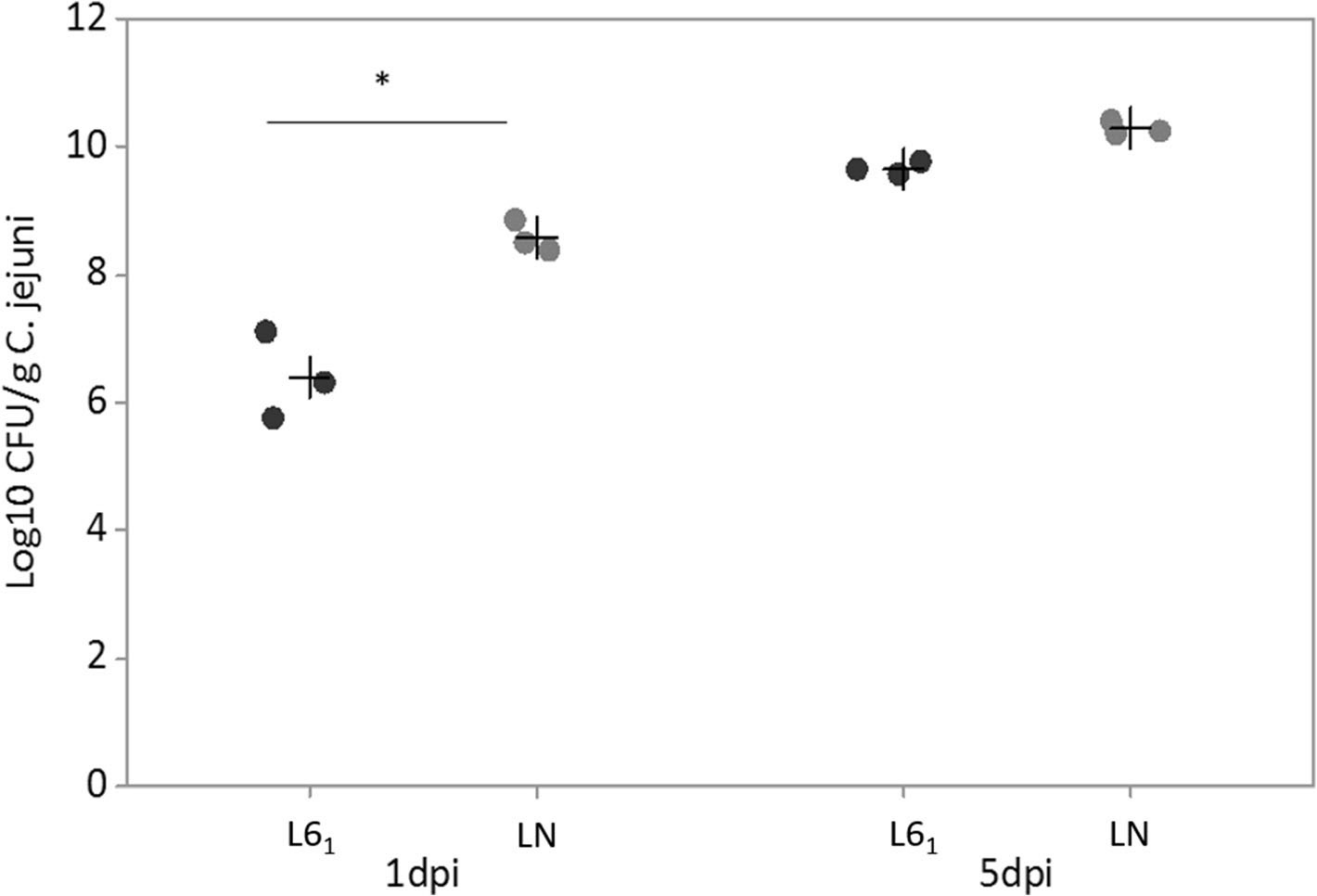
*C. jejuni* M1 colonisation in line 6_1_ and line N birds. Birds were orally inoculated at 3 weeks of age with 10^8^ CFU of *C. jejuni* M1 and the number of *C. jejuni* M1 per gram of caecal content determined at 1 and 5 dpi. Shown are the log_10_ CFU of *C. jejuni* per gram of caecal content from individual birds. *N* = 3 for each line at each time point. Crosshairs represent the mean count for each group. Significant differences were determined by Anova where * indicates significance at *P* < 0.01

To examine the level of resistance and susceptibility of lines 6_1_ and N to colonisation by *C. jejuni* M1, three-week-old birds from each line were challenged with 10^8^ CFU of *C. jejuni* M1 and the resulting numbers of *C. jejuni* in the caecal content determined at 1 and 5 days post-infection (dpi). At 1 dpi, line 6_1_ birds exhibited a significantly (*P* < 0.01) lower level of *C. jejuni* colonisation in the caeca compared to line N by approximately 3 log_10_ CFU/g (Fig. 1). At 5 dpi, no significant difference in caecal colonisation by *C. jejuni* was observed between the two lines. These results indicate that line 6_1_ is relatively resistant to *C. jejuni* M1 during early colonisation, as reported for other strains [20, 21, 29].

### Transcriptional responses to infection in chicken lines differing in *C. jejuni* resistance

To explore transcriptomic differences underlying the relative resistance of line 6_1_ and susceptibility of line N to *C. jejuni* M1 colonisation, RNA-Seq analysis was performed on caecal tonsil tissue from both infected and age-matched uninfected control birds of both lines*.* Birds were inoculated with *C. jejuni* at 3 weeks-of-age for parity with earlier reports on differential resistance to *C. jejuni* at this age [20, 21]. Differentially expressed genes (DEGs) were identified between experimental groups as follows: (1) line N infected vs control birds at 1 dpi, (2) line N infected vs control birds at 5 dpi, (3) line 6_1_ infected vs control birds at 1 dpi, (4) line 6_1_ infected vs control birds at 5 dpi and (5) line N vs line 6_1_ control birds from both 1 and 5 dpi due to the high similarity between control samples across both time points, identified by sample clustering analysis. Gene Ontology (GO) analysis using GSEABase [30] and Ingenuity Pathway Analysis (IPA) [31] were used to identify enriched gene sets and their roles in biological systems.

#### Line N transcriptional responses

Despite the high levels of caecal *C. jejuni* M1 colonisation observed in susceptible line N, only 8 and 3 DEGs were identified between infected and control birds at 1 and 5 dpi respectively (Table 1). At 1 dpi, all 8 DEGs were upregulated in infected compared to control birds whereas at 5 dpi, 2 DEGs were upregulated and 1 downregulated. DEGs relating to immune function included Interleukin 1 Receptor Like 1 (*IL1RL1*) and the C-C motif chemokine 7 (*CCL7*)*,* which were both upregulated in infected compared to control line N birds at 1 and 5 dpi, respectively. Other DEGs detected in line N were involved in cell growth and survival such as Sestrin 2 (*SESN2*) and GTPase, IMAP Family Member 8 (*GIMAP8*), which were both upregulated in infected birds. Overall, RNA-Seq analysis revealed that *C. jejuni* colonisation in line N birds produced very limited changes in gene expression.

**Table 1:** DEGs between control and infected susceptible line N birds at 1 and 5 dpi

	Gene ID	Gene name	FC	***P*** Value	FDR
DE at 1 dpi	ENSGALG00000005648	SESN2	2.06	6.86E-07	3.49E-03
	ENSGALG00000041202	FBXO32	1.98	4.56E-07	3.49E-03
	ENSGALG00000016785	IL1RL1	1.74	6.12E-06	1.27E-02
	ENSGALG00000008885	PDE1A	1.52	8.40E-07	3.49E-03
	ENSGALG00000004058	GPR146	1.51	2.07E-06	6.88E-03
	ENSGALG00000008050	HBP1	1.41	5.72E-07	3.49E-03
	ENSGALG00000008107	IRS4	1.39	3.62E-06	8.60E-03
	ENSGALG00000013489	CCDC82	1.37	3.33E-06	8.60E-03
DE at 5 dpi	ENSGALG00000041079	CCL7	9.58	3.19E-06	2.23E-02
	ENSGALG00000044062	GIMAP8	3.9	1.30E-06	2.16E-02
	ENSGALG00000031227	ELP6	0.53	4.02E-06	2.23E-02

Due to the limited number of DEGs identified between control and infected line N birds, functional annotation analysis was performed on all 8 DEGs combined from both time points. GO term enrichment analysis did not identify any enriched gene sets in the caecal tonsils of line N birds following *C. jejuni* colonisation probably due to the limited number of DEGs. IPA identified molecular functions associated with the DEGs between infected and uninfected line N birds, with pathways involved in cell death and survival, cell to cell signalling and interaction and cellular function and maintenance being the most significant (Additional File 1**: Fig. S1A**). IPA also identified a significant network of genes involved in inflammatory responses (Additional File 1**: Fig. S1B**), indicating that *C. jejuni* may elicit a limited inflammatory response in susceptible line N.

#### Line 6_1_ transcriptional responses

At 1 dpi, 69 DEGs were identified between infected and control line 6_1_ birds. Of these, 38 were upregulated and 31 were downregulated in *C. jejuni*-infected birds compared to controls (Additional file 2: **Table S1**). Genes involved in the activity of macrophages (including *MIP1a* and *MPEG1*), natural killer (NK) cells and CD8α^+^ T lymphocytes (including *EOMES*, *PRF1*, *CCL1*, CD8α chain-like 3 (*ENSGALG00000032967*), CD8α-like (*ENSGALG00000044720*)) were amongst those with the highest increase in expression in infected compared to control birds, indicating that early *C. jejuni* colonisation may stimulate inflammatory and/or antimicrobial responses in which these cell populations play a role.

Genes with the greatest reduction in expression following *C. jejuni* colonisation in line 6_1_ included members of the solute carrier family (*SLC4A9*, *SLC26A4*, *SLC51B*), G protein coupled receptor 6 member A (*GPRC6A*), TBC1 Domain Family Member 24 (*TBC1D24*), H6 Family Homeobox 2 (*HMX2*) and fibroblast growth factors (*FGF19* and *FGFBP1*). At 5 dpi, no DEGs were identified between infected and uninfected line 6_1_ birds, despite the high levels of *C. jejuni* colonisation observed. None of the identified DEGs were shared between the two lines.

GO enrichment analysis of DEGs between infected and control line 6_1_ birds at 1 dpi identified 10 associated GO terms, seven of which were upregulated in infected birds. Immune-related GO terms associated with DEGs identified included ‘Negative regulation of IL-17 Production’, ‘Chemokine Activity’ and ‘Interleukin 1 production’, all of which were upregulated in response to *C. jejuni* colonisation (Additional File 3**: Table S2**). Of the three GO terms downregulated in response to *C. jejuni* colonisation, all were involved in nucleotide transport and processing.

**Fig. 2:**
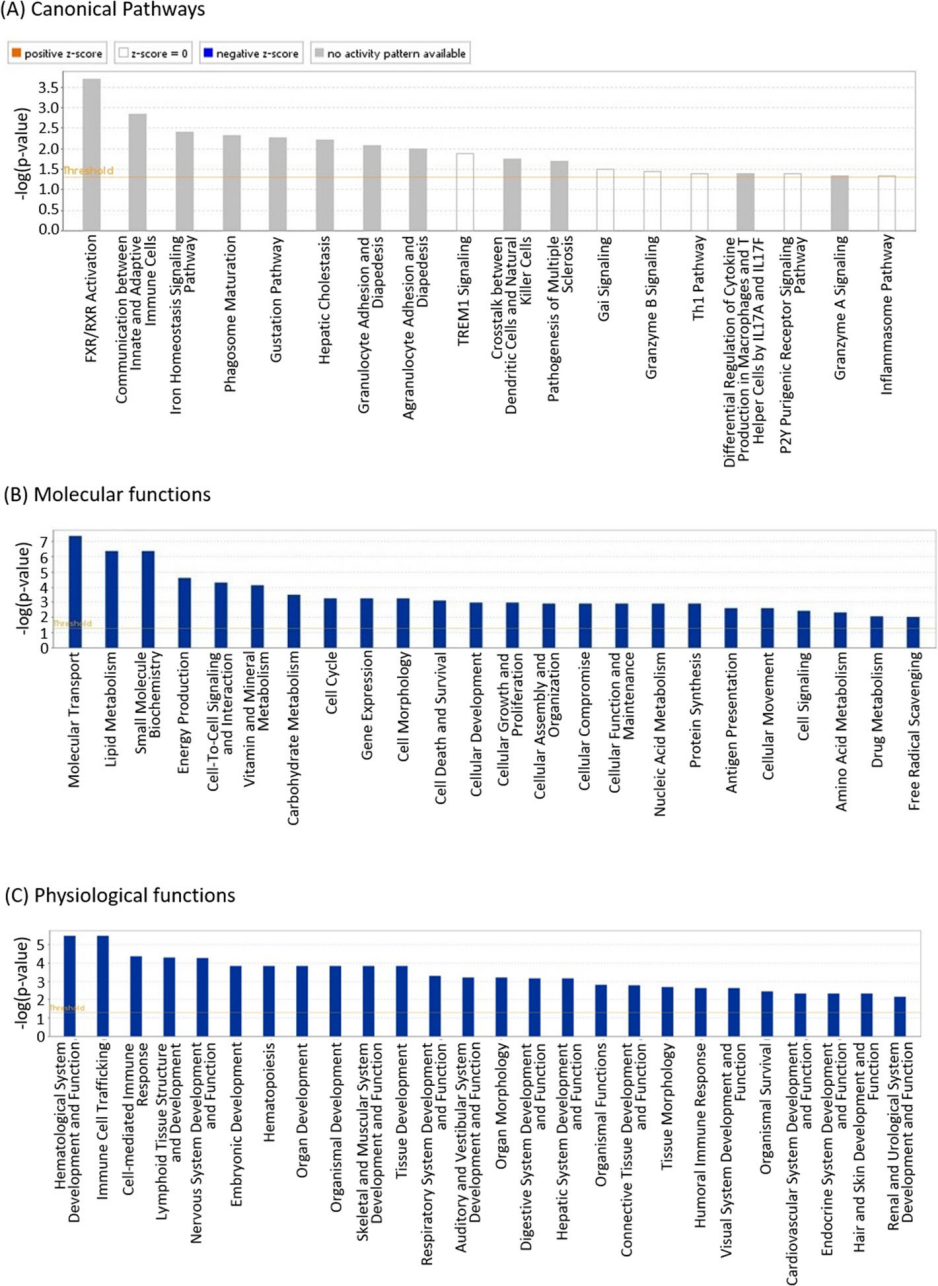
IPA of DEGs in the caecal tonsils of control and *C. jejuni* M1 colonised line 6_1_ birds at 1 dpi. Shown are the significant canonical pathways (**A**), molecular functions (**B**) and physiological functions (**C**) associated with DEGs. *N* = 3 for both groups

By IPA 18 canonical pathways associated with DEGs were identified, of which 11 were immune-related (Fig. 2A). Some of the most significant of these included ‘Communication between Innate and Adaptive Immune Cells’, ‘Phagosome Maturation’, ‘Granulocyte Adhesion and Diapedesis’, ‘Agranulocyte Adhesion and Diapedesis’, ‘TREM1 signaling’ and ‘Crosstalk between Dendritic Cells (DC) and Natural Killer Cells’. Other canonical pathways linked to resistance in line 6_1_ at 1 dpi included the FXR/RXR Activation and Iron Homeostasis signalling pathways. A number of molecular functions were identified as being significant to resistance in line 6_1_ birds following *C. jejuni* colonisation, the most significant including pathways concerning ‘Molecular Transport’, ‘Lipid Metabolism’ and ‘Small Molecule Biochemistry’ (Fig. 2B). Of the physiological functions found to be significantly related to *C. jejuni* resistance in line 6_1_, the most significant were related to immune function and included ‘Hematological System Development and Function’, ‘Immune Cell Trafficking’, ‘Cell-Mediated Immune Responses’, ‘Lymphoid System Development and Function’ and ‘Hematopoesis’ (Fig. 2C). IPA network analysis identified two significant networks of genes, involved in the antimicrobial response and cellular movement (Additional File 4**: Fig. S2A**) and lipid metabolism and transport (Additional File 4**: Fig. S2B**).

#### Comparative analysis of responses between lines after *C. jejuni* infection

**Fig. 3:**
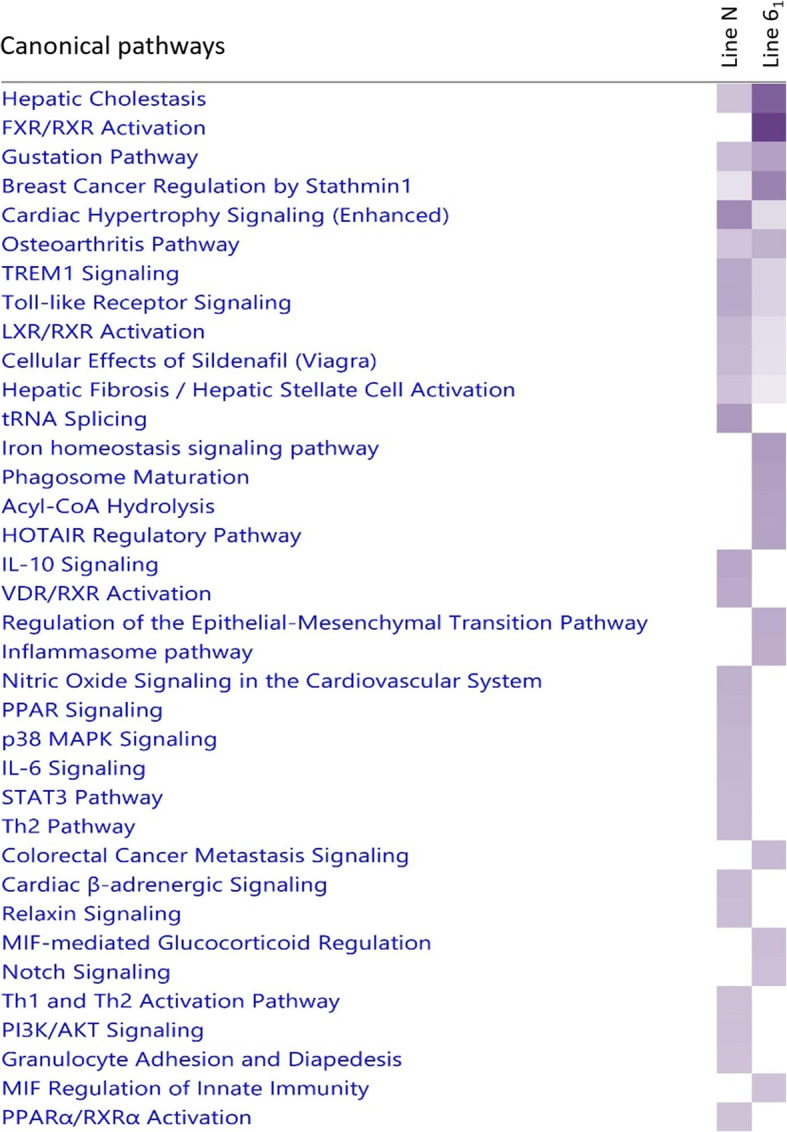
IPA comparison analysis of DEGs identified between line 6_1_ and N *C. jejuni* M1 colonised birds at 1 dpi. Shown are significant canonical pathways identified from a comparison of DEGs between 3 infected compared to 3 control birds of each line. The degree of difference in expression is denoted by the depth of colour, with a darker colour indicating a greater degree of expression

To compare differences in pathway activation in response to *C. jejuni* colonisation between the two lines, an IPA comparison was performed between activated pathways in infected birds of each line at 1 dpi (Fig. 3). A number of immune-related pathways were found to be active in line 6_1_ birds at 1 dpi, but not in line N birds, including pathways involved in macrophage activity such as ‘Phagosome Maturation’, ‘MIF-mediated Glucocorticoid’, ‘MIF Regulation of Innate Immune Responses’, and the ‘Inflammasome Pathway’. In contrast, pathways linked to Th2 (‘IL-10 Signalling’, the ‘Th2 pathway’) and IL-6 responses (‘STAT3 Pathway’ and ‘IL-6 Signalling’) were activated in line N but not line 6_1_ at 1 dpi with *C. jejuni.* With few DEG identified in line N, the same genes may underlie the pathways related to these responses. Pathways mainly involved in regulating bile and cholesterol in the liver, but which are also relevant to intestinal inflammation, were also activated to different extents in the caecal tonsils of the two lines at 1 dpi. These included the ‘FXR/RXR Activation’, ‘Hepatic Cholestasis’ and the ‘Iron Homeostasis Signalling’ pathways which were more active in line 6_1_ and the ‘LXR/RXR activation’, ‘VDR/RXR activation’ and ‘PPAR signalling’ pathways which were more active in line N. These results indicate inherent differences in the regulation of immune pathways during the early stages of *C. jejuni* infection, which may have implications for *C. jejuni* colonisation of the caeca. Significant molecular functions were also associated with the DEGs between infected birds of the two lines, including those involved in lipid and amino acid metabolic pathways (Additional File 5**: Fig. S3A**). We also identified a significant network of genes, mainly expressed to a higher degree in line N, relating to endocrine pathways (Additional File 5**: Fig. S3B**).

### Transcriptome comparison of uninfected line 6_1_ and line N birds

#### Gene expression

To investigate inherent differences between chicken lines 6_1_ and N, caecal tonsil transcriptomes were compared between control birds from each line. In total, 948 DEGs were identified between control birds of the two lines, pooled from both time points, of which 528 were more highly expressed in line N compared to line 6_1_ (Additional File 6**: Table S3**). Genes with the highest level of expression in line N compared to line 6_1_ included Histone Cluster 1 H4 Family Member D (*HIST1H4D*), Ornithine Carbamoyltransferase (*OTC*), Choline O-Acetyltransferase (*CHAT2*), CD8 alpha chain-like (*ENSGALG00000045876*) and GTPase, IMAP Family Member 5-like (*GIMAP5L*). Several genes of the major histocompatibility complex I (MHCI) were also expressed to a greater extent in line N, including *MHCIA1*, *MHCBL2* and *MHCIY*. Mucin 2 (*MUC2*), β-defensin 10 (*AvBD10*) and granzyme A (*GZMA*) were also expressed at a significantly higher level in susceptible line N. Interestingly, two genes identified in the QTL regions associated with *C. jejuni* colonisation in these lines were expressed at higher levels in line N. Acid Sensing Ion Channel Subunit Family Member 4 (*ASIC4*) was present in the QTL region on Chromosome 7 whereas *ENSGALG00000028367*, a zinc finger protein, was in the QTL identified on Chromosome 16 [21].

Of the DEGs identified between line 6_1_ and N birds, 420 genes were expressed at higher levels in line 6_1_ compared to line N. Of these, those with the greatest fold-change in expression included Class I histocompatibility antigen, F10 alpha chain-like (*LOC107050538*), Forkhead Box M1 (*FOXM1*), adenylate cyclase 5 (*ADCY5*), Deleted In Malignant Brain Tumors 1 (*DMTB1*), BPI Fold Containing Family B Member 3 (*BPIFB3*). Several other genes more highly expressed in line 6_1_ included the macrophage marker CD163-like protein (*DMBT1L*), glutathione peroxidase 2 (*GPX2*; involved in protection against oxidative stress), and trefoil factor 2 (*TFF2*; involved in stabilisation of the mucosal layer and healing of the epithelial layer).

#### Functional analysis

GO enrichment analysis performed on DEGs between the control birds of each line identified 10 associated GO terms, five of which were enriched in each line and some of which had immune function (Additional File 7**: Table S4**). Immune-related GO terms enriched in line 6_1_ compared to line N included the ‘Detection of Molecules of Bacterial Origin’, ‘Negative Regulation of IL-1β Production’ and ‘Negative Regulation of Hematopoietic Progenitor Cell Differentiation’ whereas GO terms enriched in line N compared to line 6_1_ included ‘Negative Regulation of Viral Release from Host Cell’ and ‘Negative Regulation of Leukocyte Chemotaxis’, indicating that these chicken lines may be in different states of immune readiness prior to their interactions with pathogens.

**Fig. 4:**
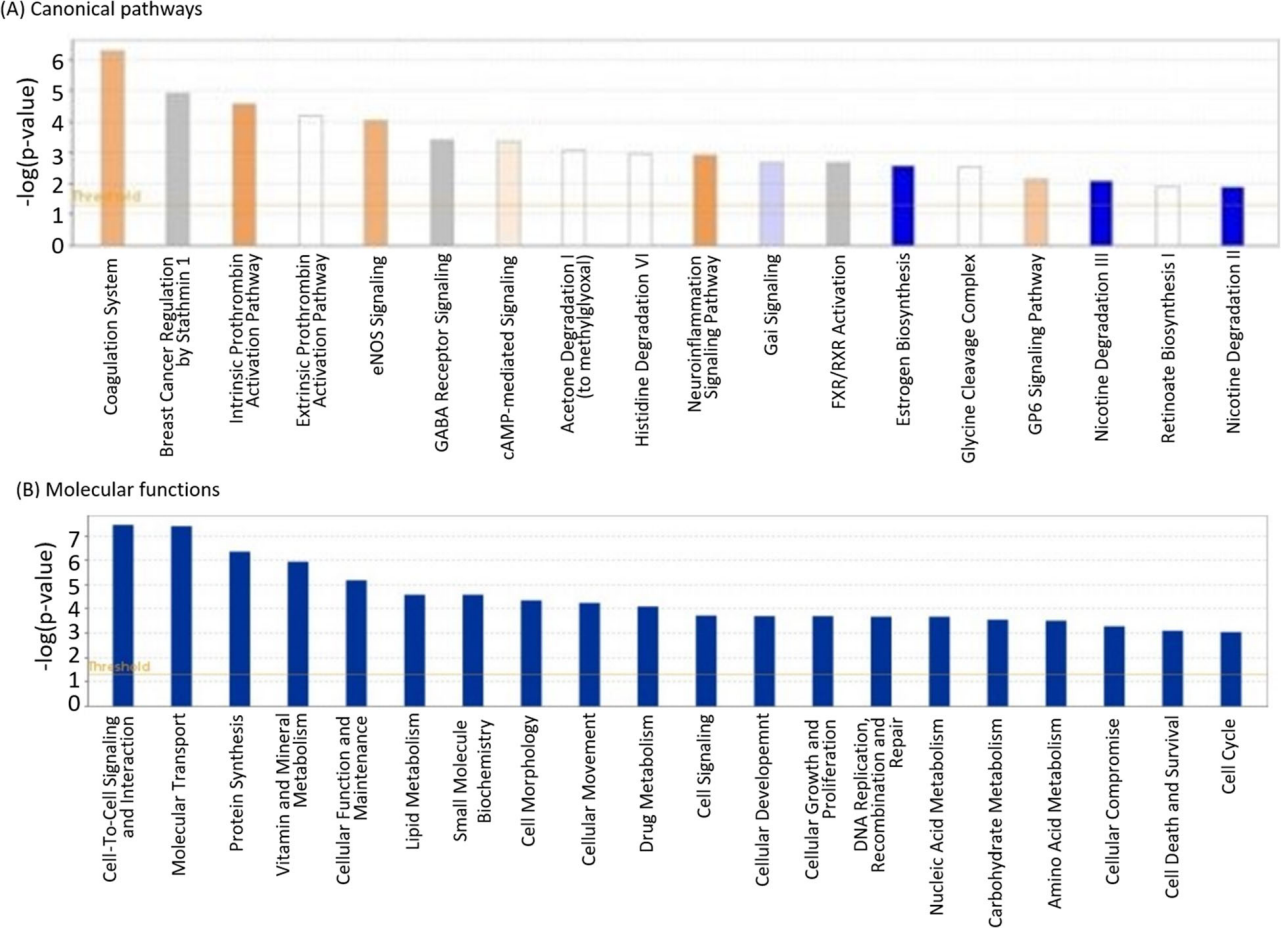
IPA of DEGs identified between control birds of line 6_1_ and N. Shown are significant canonical pathways (**A**) and molecular functions (**B**) associated with DEGs between control birds. In (**A**), red bars indicate canonical pathways upregulated in line N and blue bars indicate pathways upregulated in line 6_1_. *N* = 6 for each line (3 control birds pooled from each time point)

IPA further identified inherent differences in the level of activity of canonical pathways between the two lines (Fig. 4A). Blood coagulation pathways were more activated in line N, and included the ‘Coagulation System’ and ‘Intrinsic Prothrombin Pathway’. The ‘eNOS signalling’ pathway was also more activated in line N. Pathways more active in resistant line 6_1_ included ‘Estrogen Biosynthesis’ and ‘Nicotine Degradation II and III’. IPA also identified significant differences in molecular functions, with the most significant being ‘Cell-to-Cell Signalling and Interaction’, ‘Molecular Transport’ and ‘Protein Synthesis’ (Fig. 4B).

Significant networks of genes associated with cell-to-cell signalling (Additional File 8**: Fig. S4A**), gastrointestinal pathways (Additional File 8**: Fig. S4B**) and amino acid (Additional File 8**: Fig. S4C**) and lipid metabolism (Additional File 8**: Fig. S4D**) were identified with higher activity in line N compared to line 6_1_, highlighting that these two lines may be in different metabolic states prior to *C. jejuni* challenge and susceptibility to *C. jejuni* in line N may be due in part to distinct metabolism. Furthermore, some genes potentially acting as upstream regulators of DEGs were found to be significantly upregulated in line N, including the B-cell receptor (BCR) (Additional File 9**: Fig. S5A**), microRNA mir155 **(**Additional File 9**: Fig. S5B**) and the nuclear factor of activated T-cells (NFAT) (Additional File 9**: Fig. S5C**).

#### Gene cluster analysis

**Fig. 5:**
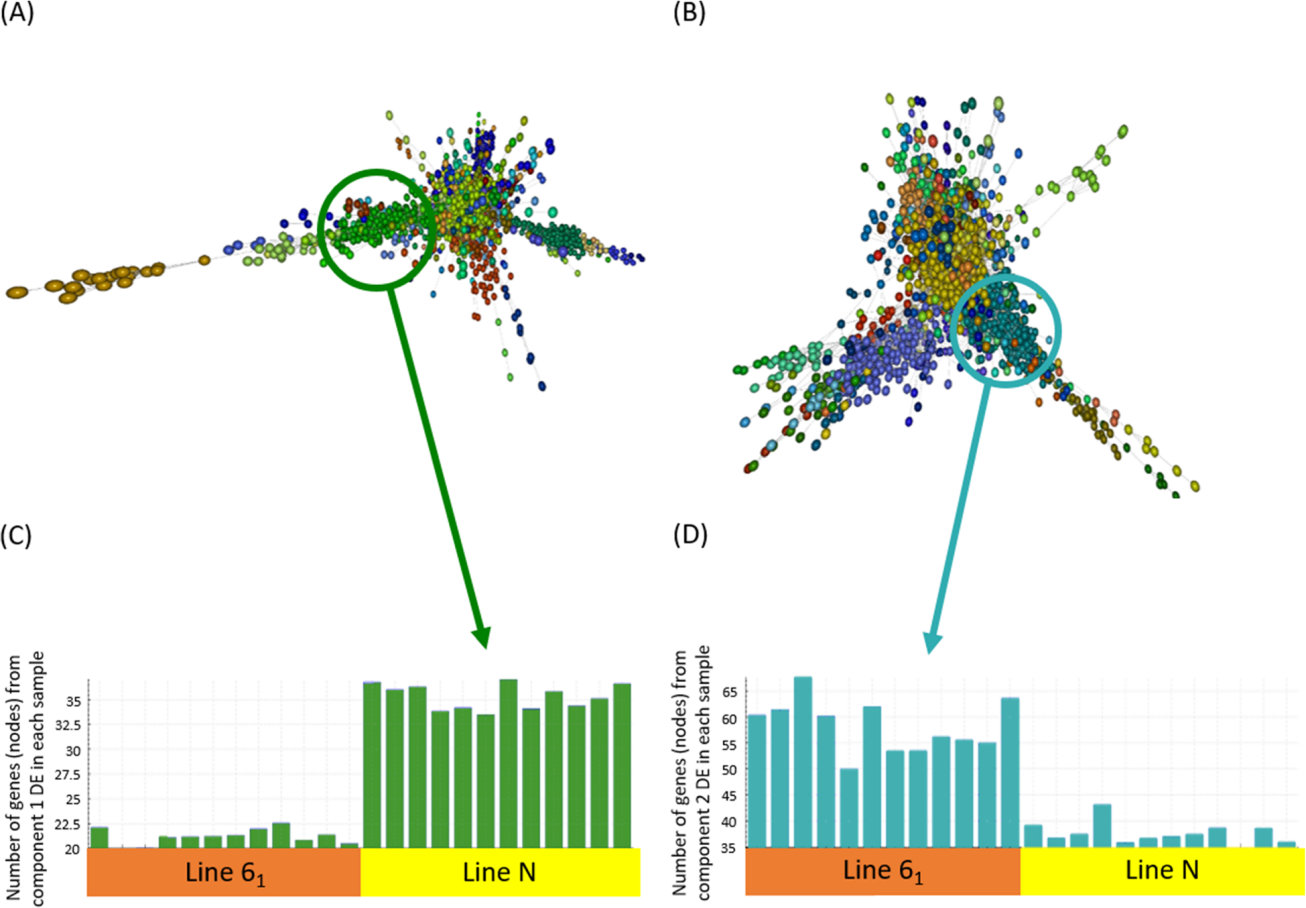
Graphia clustering of RNA-Seq data across all samples. Each node (circle) in the graph represents a gene, and each edge (line) a correlation exceeding a threshold (rho ≥ 0.93). MCL cluster granularity was set to 1.5. The most prominent clustering is by bird line with two main components being identified: component 1 - genes expressed at higher levels in line N (**A**) and component 2 - genes expressed at higher levels in line 6_1_ (**B**). Colours indicate clusters of genes which have a similar expression pattern. (**C**) and (**D**) show the number of genes from each cluster in individual birds, identified by matching colours in corresponding components

Graphia software [32] analysis revealed the most prominent clustering was by bird line, suggesting that basal gene expression differences between lines 6_1_ and N may explain intrinsic resistance as opposed to differences in their response to *C. jejuni* infection. Two components containing the majority of DEGs were identified. These were Component 1 comprising of 2822 genes expressed to a greater extent in line N and Component 2 comprising of 2285 genes expressed to a greater extent in line 6_1_ (Fig. 5A and B **respectively**). Mean histogram plots of all genes present within these two components indicated that genes were generally expressed at higher levels in one line compared to the other indicating major differences in the regulation of groups of genes are key to the resistance and susceptible phenotypes in these lines (Fig. 5C and D).

### Validation of DEGs by qRT-PCR

**Fig. 6:**
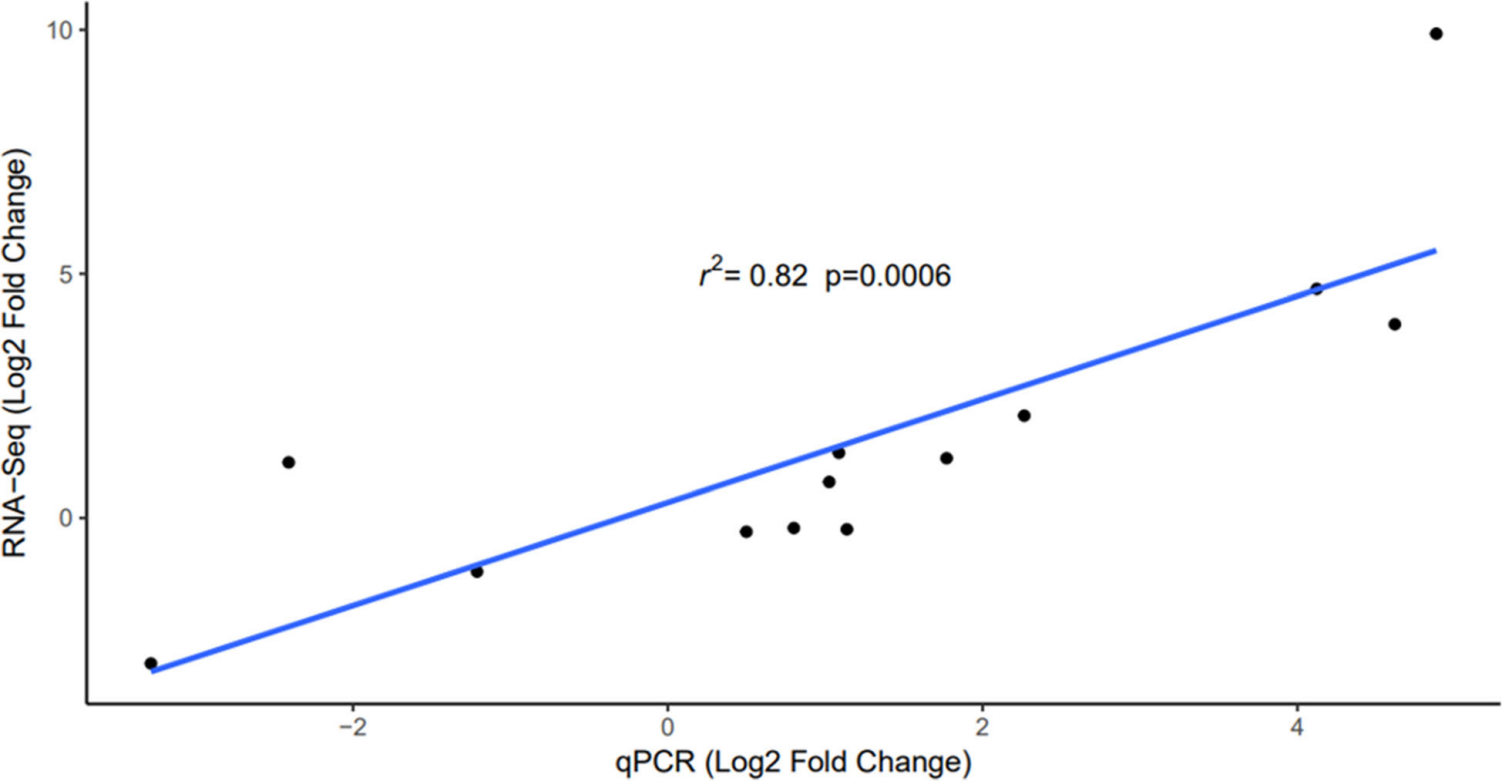
qRT-PCR validation of DEGs identified by RNA-Seq. Shown is the correlation of the log_2_ fold change in mRNA transcripts as determined by RNA-Seq (y-axis) with the log_2_ fold change of mRNA transcripts as determined by qRT-PCR (x-axis). DEGs that were validated are given in Additional file 11: Table 6. The line of best fit is represented in blue with the R^2^ value denoted on the graph

RNA-Seq data was validated by qRT-PCR analysis of a subset of genes. These were chosen for validation based on their possible biological significance during *C. jejuni* colonisation and the degree to which they were DE. Genes were mainly selected from the pairwise comparison between control birds of each line, owing to the high number of DEGs identified in this group. Correlation of the qRT-PCR results with the RNA-Seq results produced a correlation co-efficient of R^2^ = 0.86 (*p* < 0.001) therefore the qRT-PCR results are comparable to the RNA-Seq data (Fig. 6).

## Discussion

With the handling or consumption of contaminated chicken accounting for a high proportion of human campylobacteriosis [6, 7], a pressing need exists to reduce the prevalence of *C. jejuni* in commercial broiler flocks. White Leghorn inbred lines 6_1_ and N have an established difference in heritable resistance to *C. jejuni* colonisation, with genetic variation associated with *C. jejuni* resistance identified by genome-wide association studies using backcross and advanced 6_1_ x N intercross populations [21]. Furthermore, key QTL associated with resistance to *C. jejuni* are shared between these inbred lines and commercial broilers [14] indicating that findings in inbred chickens can be highly relevant to *C. jejuni* control in commercial flocks. Using RNA-Seq, we evaluated transcriptomic differences in the caecal tonsils of these inbred lines, both inherently and following *C. jejuni* colonisation, to investigate the basis of their differential resistance to *C. jejuni* and potentially obtain biomarkers that could be selected in commercial populations.

We showed that compared to line N, line 6_1_ was relatively resistant to early *C. jejuni* M1 colonisation in the caeca at 1 dpi, however by 5 dpi both lines were similarly susceptible to colonisation. Previously, Boyd et al reported resistance to cloacal and caecal colonisation was apparent in line 6_1_ compared to line N from 4 to 20 dpi [20], a discrepancy with this study likely due, in part, to the different *C. jejuni* strains used by Boyd (*C. jejuni* 14 N and 81–176) and in this report (*C. jejuni* M1). Chintoan-Uta et al recently reported line N birds to be colonised by *C. jejuni* 11168H at c. 10^4^ CFU/g caecal contents 9 dpi at 3 weeks-of-age whereas the challenge strain was absent at the limit of detection by direct plating at this time in line 6_1_ birds [29]. M1 rapidly colonises the chicken caeca from doses as low as 100 CFU [33, 34] and both the rate of colonisation and caecal burden have proven greater than for other *C. jejuni* strains tested in the same model, including 11168H (Stevens et al, unpublished data). For many *C. jejuni* strains, there is a minimum infective dose which can cause high levels of colonisation in the chicken caeca [35–37]. It is plausible that both chicken lines may have been overwhelmed by the dose of *C. jejuni* M1 administered, now known to be considerably higher than the minimum dose required for reliable colonisation (10^2^ CFU, [33]).

Susceptible line N displayed a very limited caecal transcriptional response to *C. jejuni* colonisation compared to resistant line 6_1_ where a wider, albeit brief, response at 1 dpi was observed. Although functional annotation analysis identified significant pathways related to immune function in both lines, the difference in magnitude between the responses of the two lines may partly explain the relative resistance and susceptibility displayed at 1 dpi. DEGs related to macrophage, NK cell and CD8α^+^ T cell activity were upregulated in resistant line 6_1_ at 1 dpi, with functional annotation analysis identifying pathways involved in communication between the innate and adaptive arms of immunity, phagosome maturation and crosstalk between NK cells and DC as involved. It follows that the lower level of colonisation in line 6_1_ at 1 dpi may, in part, be attributable to this innate response whereas at 5 dpi, this response is absent and coincides with line 6_1_ caecal colonisation levels matching those of line N. It is possible that NK cells are involved in the initial response to *C. jejuni* observed in line 6_1_ as the NK complex is located in close proximity to the chicken MHC B complex, which influences the responsiveness of chicken NK cells [38], and several DE MHCI genes were identified between lines N and 6_1_, including *MHCIA1*, *MHCBL2* and *MHCIY.*

Innate inflammatory responses have been linked to reduced *C. jejuni* colonisation elsewhere. Although not detected in our study, Psifidi et al found *CXCLi1* and *CXCLi2* (proinflammatory chemokines involved in heterophil chemotaxis) expression was reduced in the caecal tonsils of both line 6_1_ and N following *C. jejuni* infection [21], but more so in line N, further implying innate responses may be involved in controlling *C. jejuni* colonisation at this site. Moreover, broilers selected for an inflammatory phenotype (high levels of IL-6, CXCLi2 and CCLi2) are less likely to become colonised by *C. jejuni* compared to those selected for lower inflammatory phenotype [22], indicating that an inherent proinflammatory status reduces the ability of *C. jejuni* to colonise the chicken caeca. However, in contrast, Humphrey et al found that levels of *CXCLi1* and *CXCLi2* expression were not related to differences in caecal *C. jejuni* load [27]*.*

Iron homeostasis signalling pathways were upregulated in resistant line 6_1_ at 1 dpi. Iron is essential for bacterial replication and mutants with defects in iron acquisition are frequently attenuated [39], therefore differences in iron availability in the gastrointestinal tract between chicken lines may factor in their relative resistance and merits further investigation. Additionally, higher activity related to MIF-mediated glucocorticoid, the MIF innate immune response and inflammasome pathways occurred during initial *C. jejuni* colonisation in line 6_1_, indicating that MIF and the inflammasome may be mediators in the initial inflammatory response in line 6_1._

Major transcriptional differences were apparent between control birds of both lines, and were far greater than those observed in response to *C. jejuni* infection. Our study is not the first to identify such a degree of inherent transcriptional variation between White Leghorn inbred lines. For example, gene expression in the spleen and thymus differs by several hundred genes between lines 6_1_ and 7_2_, which are relatively resistant and susceptible to Marek’s Disease Virus respectively [40]. Significant networks of genes associated with amino acid and lipid metabolism were upregulated in line N control birds, consistent with other studies linking higher inherent metabolic states with susceptibility to *C. jejuni*. Li et al (2010) found amino acid, lipid and glucose metabolic pathways to be upregulated in a *C. jejuni-*susceptible line compared to a resistant line following colonisation, with amino acid processes also upregulated in control birds of the susceptible line [15]. Additionally, Li et al (2011) found increased expression of genes involved in fatty acid and protein metabolic processes between *C. jejuni-*colonised compared to non-colonised birds of the same line [16]. Our data identified differences in transcription of genes associated with metabolic activity between the lines coinciding with differential colonisation levels at 1 dpi. For example, higher activation in the farnesoid x receptor (FXR)/retinoid x receptor activation was present in line 6_1_ but liver x receptor (LXR)/RXR, vitamin D receptor (VDR)/RXR and peroxisome proliferator-activated receptor (PPARα)/RXR activation was higher in line N. FXR, LXR and PPAR-α are mainly associated with lipid metabolism and in the regulation of triglyceride levels in mammals [41–43]. However, between them, FXR, LXR and PPAR-α also have various roles in regulating intestinal inflammation and immunity including effects on macrophage inflammatory activity, reducing the presence of reactive oxygen species (ROS) and maintaining the intestinal barrier [44–49], and are considered potential therapeutic targets in the case of mammalian IBD [45, 50, 51]. Expression of FXR, LXR and PPAR-α in these chicken lines may therefore modulate intestinal inflammatory responses, influencing *C. jejuni* colonisation. Furthermore, their roles in triglyceride metabolism may also influence colonisation as triglyceride-rich lipoprotein can bind LPS, reducing LPS toxicity during bacterial infection and reducing macrophage activation [52]. Vitamin D and its receptor are important in maintaining the mucosal barrier of the intestine [53] and modulate proinflammatory cytokine production [54]. Abiotic *IL10*^−/−^ mice treated with artificial vitamin D suffered less diarrhoea and had lower levels of intestinal IL-6, IFN-γ and CCL2 during campylobacteriosis than mice that did not receive vitamin D [55], therefore the contribution of vitamin D and the vitamin D receptor to *C. jejuni* susceptibility in line N chickens warrants further study. Upstream gene regulators involved in several immune-related networks were associated with the DEGs identified between control birds of both lines (BCR, NFAT and miR-155). Previously, increased caecal miR-155, a regulator of inflammatory processes in mammals [56], was observed following *C. jejuni* colonisation in chickens [57]. These differences in inflammatory states prior to *C. jejuni* inoculation likely influence the speed and resulting level of colonisation in different chicken lines.

Lines 6_1_ is also relatively resistant to *S.* Typhimurium colonisation compared to line N [28, 58]. QTLs associated with resistance to both *S.* Typhimurium and *C. jejuni* have been identified on chromosome 14 [21, 59] and chromosome 16 [21, 60, 61] implying that similar mechanisms of genetic control may confer resistance to both *C. jejuni* and *Salmonella.* Furthermore, greater inflammatory responses to *Salmonella* have been observed in resistant chicken lines compared to susceptible birds [62] highlighting the importance of innate responses in resistance to enteric bacterial pathogens.

As *C. jejuni* mainly resides in the chicken intestinal lumen, it is plausible that differences at the mucosal surface or extracellular milieu may account for resistance. Genes encoding MUC2 and AvBD10 were expressed to a higher level in line N compared to line 6_1_ control birds. Previously, it has been shown that chicken mucus inhibits *C. jejuni* invasion of both chicken and human primary intestinal cells compared with human mucus [63]. It is therefore possible that inherent differences in the composition of chicken mucus between lines accounts for the differences in the colonisation levels observed. *GZMA* was also more highly expressed in line N, corresponding to a microarray study where *GZMA* expression was higher in the caeca of *C. jejuni-*colonised compared to non-colonised birds, indicating *GZMA* is linked with elevated *C. jejuni* levels in the caeca [16]. Recent studies have identified mostly limited differences in the microbial communities present in the caeca of lines 6_1_ and N at the age they were inoculated herein, but the extent to which these differences drive distinct patterns of gene expression in the gut, or vice versa, is unknown [29].

Other studies have examined the impact of gene expression and host genetics on *C. jejuni* colonisation of the caeca [14, 16, 17, 19, 20, 23, 24]. However, these varied in the strain of *C. jejuni* used, bird line and challenge age of the birds (between 2 and 4 weeks-of-age), and sampling intervals (from 6 h to 14 dpi) resulting in variation of the level of colonisation observed and challenges in extrapolating from one study to the next. Despite these differences, a similar narrative can be seen throughout these studies whereby a moderate number of significant DEGs was identified upon *Campylobacter* challenge, but often at a low fold-change indicating that even high levels of *C. jejuni* colonisation does not elicit a dramatic response in the chicken [16, 17, 21, 64]. We elected to study the transcriptome of caecal tonsils as a gut-associated lymphoid tissue at a key site of *Campylobacter* persistence, and to permit direct comparison with RNA-Seq data from resistant or susceptible broilers from a recent genome-wide association study [14] and earlier qRT-PCR data on candidate resistance-associated genes in lines 6_1_ and N [21]. The former study detected differentially transcribed genes in caecal tonsils within quantitative-trait loci associated with resistance to caecal colonisation, indicating that it is a relevant anatomical location to sample. However, we cannot preclude the possibility that the responses observed may not be typical of the wider caecal mucosa, or tissue at other key sites of *Campylobacter* persistence in poultry.

A GWAS on an advanced intercross of line 6_1_ and N previously identified 6 SNPs on chromosomes 4, 14 and 16 associated with resistance to *C. jejuni* colonisation [21]. We identified two genes located within the QTL regions associated with *C. jejuni* colonisation in these lines, *ASIC4* (Acid Sensing Ion Channel Subunit Family Member 4) and *ENSGALG00000028367*, both of which were inherently expressed to a greater extent in line N. *ASIC4* is broadly expressed in the mammalian nervous system but its role in birds is unknown. In mammals, ASICs are known for their role in neuroinflammation [65] and promote exocytosis and maturation in bone marrow-derived macrophages stimulated by extracellular acidosis [66]. *ENSGALG00000028367*, or *BZFP2*, is a zinc finger-like protein which likely binds nucleic acids, proteins and other small molecules [67], and bares similarity to the *CKR1*-like gene and is present in a region of the MHC-B locus [68].

To conclude, RNA-Seq analysis demonstrated an initial inflammatory response to *C. jejuni* infection of a greater magnitude in resistant line 6_1_ compared to line N, which may be associated with caecal colonisation. This response was short lived and absent at later intervals when differential resistance was not detected. By far, the most striking differences in gene expression were detected between uninfected control birds of the two lines. The identification of candidate genes involved in early innate responses and metabolic pathways provides a foundation for future studies on avian heritable resistance to *Campylobacter*.

## Methods

### Animals

Two inbred White Leghorn chicken lines were used in this study. Lines 6_1_ and N, reported to be relatively resistant and susceptible to *C. jejuni* colonisation respectively, originate from the USDA ARS Avian Disease and Oncology Laboratory (formerly the Regional Poultry Laboratory) in East Lansing, MI, USA [12]. Flocks of both lines were maintained under specified pathogen-free (SPF) conditions at the National Avian Research Facility (NARF) at the Roslin Institute, UK.

### Bacterial culture, experimental design and sample collection

*C. jejuni* strain M1 [69] was used to inoculate animals in this study. *C. jejuni* M1 was cultured on modified charcoal-cephoperazone-microaerophillic agar (mCCDA; Oxoid) or in Mueller-Hinton (MH) broth (Oxoid) at 37 °C with 5% O_2_, 5% CO_2_ and 90% N_2_ in a microaerophilic cabinet. Liquid cultures in broth were with shaking at 400 rpm under the same atmospheric conditions.

Twelve birds each of White Leghorn lines 6_1_ and N were obtained on the day of hatch and housed under SPF conditions with access to feed and water ad libitum*.* For each line there were 6 control and 6 infected birds. At 3 weeks-of-age, birds of the infected groups were challenged by oral gavage with 10^8^ *C. jejuni* M1 in 0.1 ml of MH broth and birds of the control groups given an equivalent volume of MH broth only. At 1 and 5 dpi, 3 chickens of each group were culled by cervical dislocation and death confirmed by permanent cessation of blood circulation. At post mortem examination, caeca were collected for the enumeration of *C. jejuni* by plating 100 μl of triplicate 10-fold serial dilutions of caecal contents in phosphate-buffered saline (PBS) to mCCDA plates. Caecal tonsils were collected promptly into RNA*later*® (Life Technologies) and stored at − 80 °C until processing.

### RNA-Seq library preparation, sequencing and data analysis

RNA was extracted from both caecal tonsils using the Qiagen RNeasy mini kit according to the manufacturer’s instructions. RNA preparations were assessed for quantity and quality using a Qubit™ Fluorimeter (Invitrogen) and an Agilent 2200 TapeStation (Agilent Technologies) respectively. The resulting RNA integrity number (RIN) values for all RNA samples was greater than 9 therefore all 24 samples (*n* = 3 per group) were subsequently submitted to the Edinburgh Genomics sequencing facility (Edinburgh, UK) for RNA-Seq. The TruSeq stranded mRNA-Seq Library Prep kit (Illumina) was used to generate mRNA libraries free from ribosomal RNA and the resulting transcriptomes sequenced using the Illumina HiSeq 4000 system to generate 75 bp paired end reads at a depth of 50 million reads per sample.

The quality of the raw sequence reads was assessed using FASTQC (v0.11.5) [70]. Cutadapt (v1.14) [71] was used to trim adapter sequences, remove bases with a Phred score of less than 30 and ensure resulting sequences were at least 50 bp in length. Trimmed reads were then mapped to the *Gallus gallus* reference genome (GalGal5, Ensembl release 89) using the STAR aligner software package (v2.5.1b) [72] with a minimum alignment of 52.5 million input reads for each sample (Additional File 10**: Table S5**). In all samples, over 90% of reads were uniquely mapped with fewer than 10% either unmapped or mapped to multiple loci. The number of reads aligning to genomic features were determined using FeatureCounts (v1.5.3) [73] using default parameters. Analysis of DEGs was performed in R using EdgeR package with DEGs exhibiting a fold change (FC) > 2 and a false discovery rate (FDR) < 0.05 considered significant.

Gene ontology term enrichment analysis was performed in R using the GSEABase package (downloaded from the Gene Set Enrichment Analysis (GSEA) website, https://software.broadinstitute.org/gsea/index.jsp, file: C5.all.v6.0.symbols). Significant gene ontology was determined using the mRoast function of the Limma package in R for each pairwise comparison. Ingenuity Pathway Analysis (IPA) was performed on significant DEGs using IPA software (Qiagen Bioinformatics, www.qiagenbioinformatics.com/products/ingenuity-pathway-analysis) to classify their associated biological functions, canonical pathways and biological networks. Network analysis was performed using the normalised raw counts in Graphia Pro [32] with a Pearson correlation threshold of r = 0.93. The number of nodes (genes) in the analysis was 6066 linked by 181.1 k edges. Markov Clustering (MCL) was performed with a granularity of 1.5 on these networks to identify components containing genes of similar expression patterns. Components of less than 10 nodes were removed from the analysis. Gene lists derived from Graphia were submitted to the functional annotation tool DAVID [74] to further investigate the roles of genes identified in the components, with *Gallus gallus* selected as the background for these lists.

### cDNA preparation and quantitative reverse-transcriptase polymerase chain reaction (qRT-PCR)

Genes related to immune function that were observed to be DE by RNA-Seq were validated by qRT-PCR. The Verso cDNA Synthesis kit (Thermo Scientific) was used to prepare cDNA from 1 μg of total RNA according to the manufacturer’s instructions. Quantitative PCR reactions were performed using the Forget-Me-Not™ qPCR Master Mix (Biotium) in 20 μL volumes containing 1 X Forget-Me- Not™ qPCR Master Mix, 0.5 μM each of the forward and reverse primers, 50 nM of ROX reference dye and 2 μL of cDNA at a 1:10 dilution in template buffer. Gene-specific primers were purchased from Sigma and are detailed in Additional File 11**: Table S6.** The amplification and detection of specific DNA was achieved using the AB 7500 FAST Real-Time PCR System (Applied Biosystems) to the following program: 95 °C for 2 min followed by 40 cycles of 95 °C for 5 s then 60 °C for 30 s. To confirm the presence of a single PCR product, melting curves were generated by 1 cycle of 60 °C for 1 min, increasing to 95 °C in 1% increments every 15 s. Samples were run in triplicate and each qPCR experiment contained 3 no-template controls and a 5-fold dilution series in duplicate of pooled caecal tonsil-derived cDNA from several birds from which standard curves were generated. The relative expression of genes were calculated as described by Pfaffl [75] and normalised to the geometric mean of three reference genes; *r28S*, *TBP* and *GAPDH*. A Spearman’s correlation was calculated between the log_2_ fold-change of the RNA-Seq results to the log_2_ fold-change detected by qPCR in R.

## Supplementary Information

**Additional file 1: Figure S1.** IPA of DEGs in the caecal tonsils between control and *C. jejuni* M1 colonised line N birds at 1 dpi. Shown are significant molecular functions (A) associated with DEGs and a significant network of inflammatory responses involved during *C. jejuni* infection of line N at 1 dpi (B). In (B), genes or nodes coloured red are upregulated in *C. jejuni* colonised birds whereas those in green are downregulated. *N* = 3 for both groups.

**Additional file 2: Table S1.** DEGs in the caecal tonsils between control and infected line 6_1_ birds at 1 dpi. A fold change greater than 1 indicates genes that were upregulated in infected compared to control birds whereas a fold change less than 1 indicates genes which were downregulated in infected compared to control birds. *N* = 3 for both groups. FC: fold change, FDR: False Discovery Rate.

**Additional file 3: Table S2.** Significant GO Terms associated with DEGs in the caecal tonsils between infected and control line 6_1_ birds at 1 dpi. DEGs were identified between 3 infected and 3 control birds. FDR: False Discovery Rate.

**Additional file 4: Figure S2.** IPA of DEGs in the caecal tonsils of control and *C. jejuni* M1 colonised line 6_1_ birds at 1 dpi. Shown are a significant network of genes involved in the antimicrobial response and cellular movement (A) and of genes involved in lipid metabolism and transport (B). Genes or nodes coloured red are upregulated in colonised compared to control birds whereas those in green are downregulated.

**Additional file 5: Figure S3.** IPA comparison of DEGs identified in the caecal tonsils between line 6_1_ and N *C. jejuni* M1 colonised birds at 1 dpi. Shown are significant molecular functions (A) and a significant network of genes related to endocrine pathways (B) identified from the comparison of DEGs between 3 infected and 6 control birds of each line. In (B), genes or nodes coloured red are upregulated in colonised line N birds whereas those in green are upregulated in colonised line 6_1_ birds.

**Additional file 6: Table S3.** DEGs in the caecal tonsils between line N and line 6_1_ control birds. Analysis compares the gene expression of all six control birds for each line. A fold change greater than 1 indicates genes more highly expressed in line N whereas a fold change less than 1 indicates genes more highly expressed in line 6_1_. FC: fold change, FDR: False Discovery Rate.

**Additional file 7: Table S4.** Significant GO Terms associated with DEGs in the caecal tonsils between line N and line 6_1_ control birds. Upregulated GO terms are associated with genes with higher expression in line N and downregulated GO terms are associated with genes with higher expression in line 6_1_. DEGs were identified between all six control birds of each line. FDR: False Discovery Rate.

**Additional file 8: Figure S4.** IPA of DEGs in the caecal tonsils identified between control birds of line 6_1_ and N. Shown are significant networks of genes relating to cell-to-cell signalling (A), gastrointestinal pathways (B), amino acid metabolism (C) and lipid metabolism (D). *N* = 6 for each line (3 control birds pooled from each time point) Genes or nodes coloured red are upregulated in colonised line N birds whereas those in green are upregulated in colonised line 6_1_ birds.

**Additional file 9: Figure. S5.** IPA of DEGs identified between control birds of line 6_1_ and N. Shown are significant networks associated with predicted upstream regulators of DEGs: BCR (A), mir155 (B) and NFAT (C). Genes or nodes coloured red are upregulated in line N birds whereas those in green are upregulated in line 6_1_ birds. N = 6 for each line (3 birds pooled from each time point)

**Additional file 10: Table S5.** STAR Alignment statistics for RNA-Seq transcript reads. l6: line 6_1_, ln: line N, i: infected, c: control, b: bird, dpi: days post-infection.

**Additional file 11: Table S6.** Primer sequences for qRT-PCR

## Data Availability

Sequencing data have been submitted to the European Nucleotide Archive (https://www.ebi.ac.uk/ena) under accession number PRJEB24399.

## Abbreviations

cDNA: Complementary DNA
DC: Dendritic cell
DE: Differentially expressed
DEGs: Differentially expressed genes
dpi: Days post-infection
FC: Fold change
FDR: False discovery rate
GO: Gene ontology
IPA: Ingenuity Pathway Analysis
mCCDA: Modified charcoal-cefoperazone-deoxycholate agar
NK: Natural killer
qRT-PCR: Quantitative reverse-transcriptase polymerase chain reaction
SPF: Specified pathogen-free
