# Supplementary figures and images for: Transcriptomic analysis of caecal tissue in inbred chicken lines that exhibit heritable differences in resistance to Campylobacter jejuni
Source: BMC Genomics. 2021 Jun 4;22:411. doi: 10.1186/s12864-021-07748-2 (PMC8176612; doi:10.1186/s12864-021-07748-2)

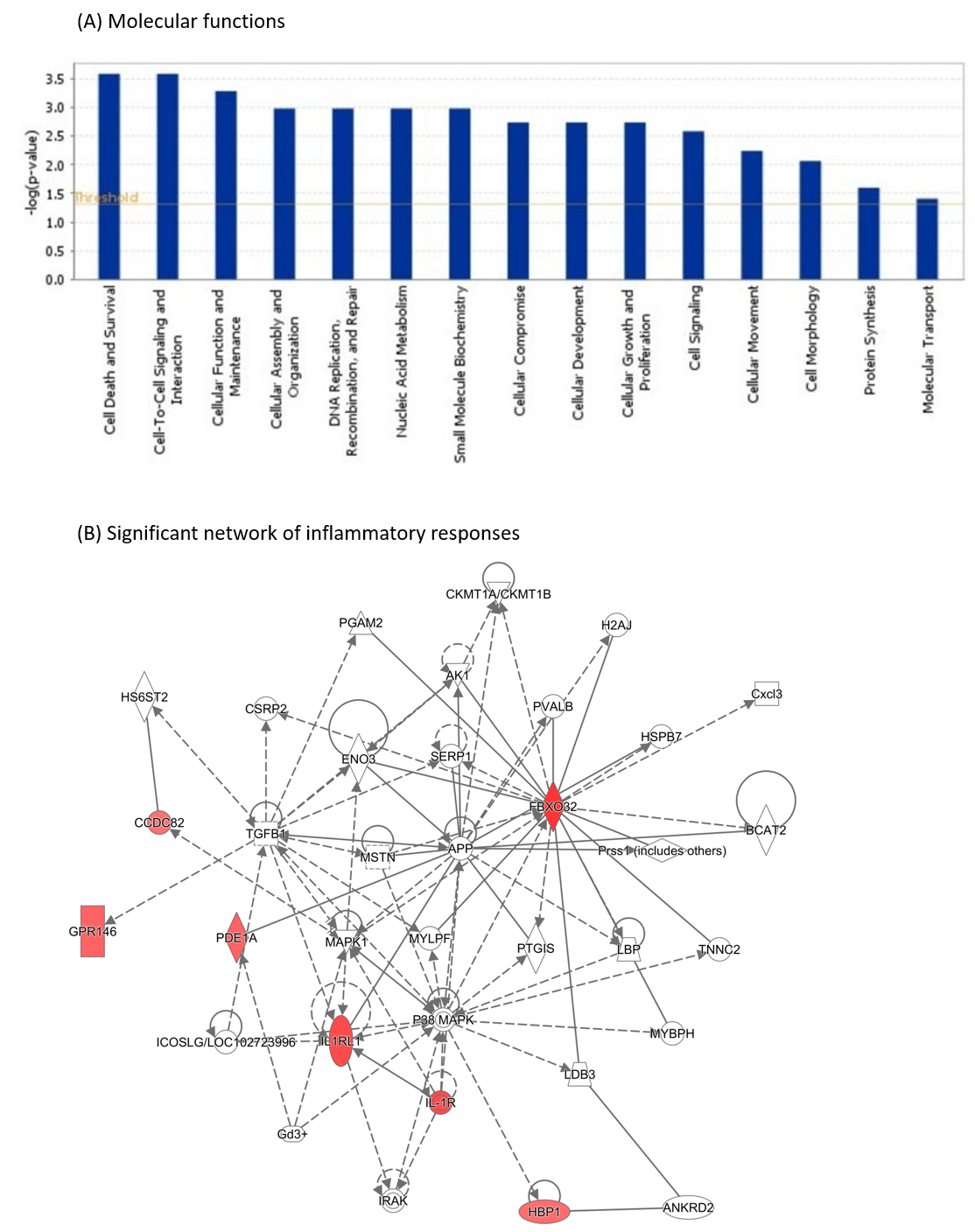

Supplement: Supplementary file 1 — Additional file 1: Figure S1. IPA of DEGs in the caecal tonsils between control and C. jejuni M1 colonised line N birds at 1 dpi. Shown are significant molecular functions (A) associated with DEGs and a significant network of inflammatory responses involved during C. jejuni infection of line N at 1 dpi (B). In (B), genes or nodes coloured red are upregulated in C. jejuni colonised birds whereas those in green are downregulated. N = 3 for both groups. [file 12864_2021_7748_MOESM1_ESM.tif]

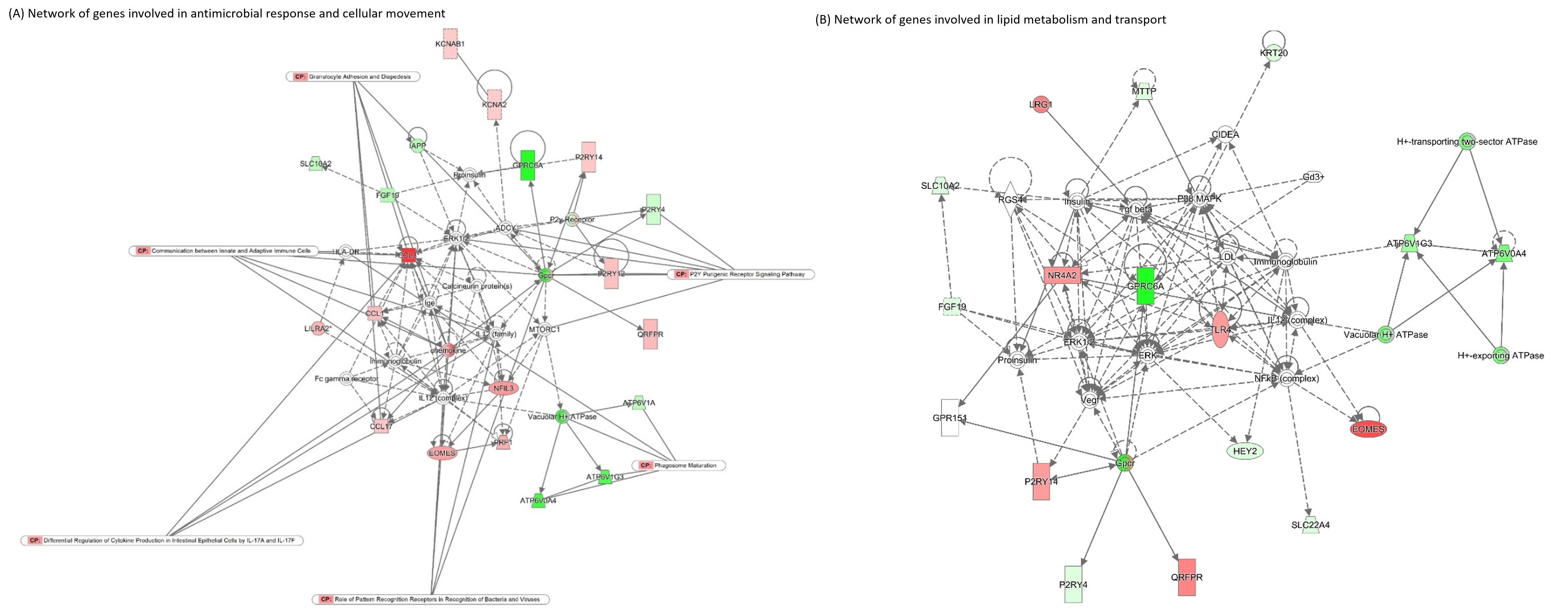

Supplement: Supplementary file 4 — Additional file 4: Figure S2. IPA of DEGs in the caecal tonsils of control and C. jejuni M1 colonised line 61 birds at 1 dpi. Shown are a significant network of genes involved in the antimicrobial response and cellular movement (A) and of genes involved in lipid metabolism and transport (B). Genes or nodes coloured red are upregulated in colonised compared to control birds whereas those in green are downregulated. [file 12864_2021_7748_MOESM4_ESM.tif]

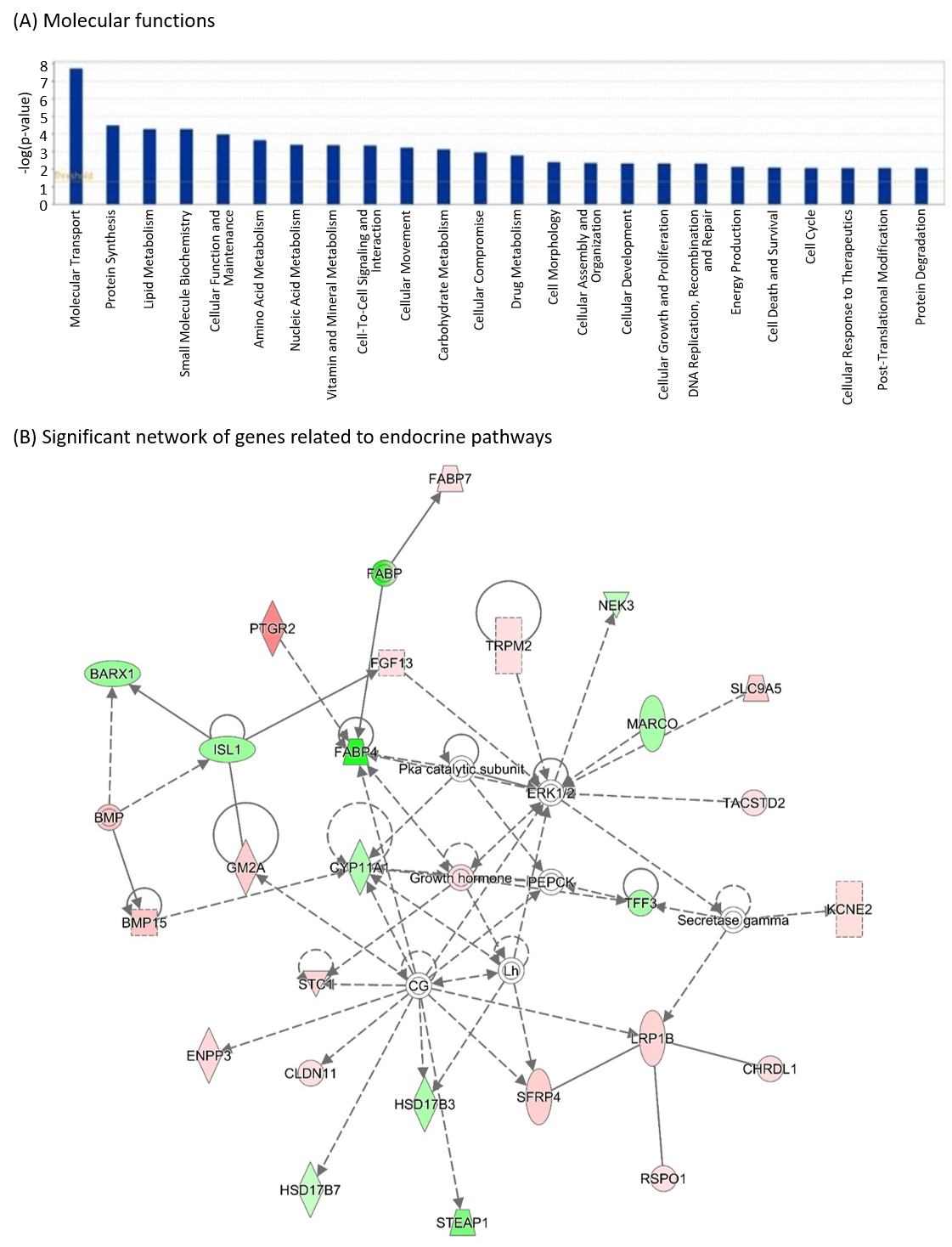

Supplement: Supplementary file 5 — Additional file 5: Figure S3. IPA comparison of DEGs identified in the caecal tonsils between line 61 and N C. jejuni M1 colonised birds at 1 dpi. Shown are significant molecular functions (A) and a significant network of genes related to endocrine pathways (B) identified from the comparison of DEGs between 3 infected and 6 control birds of each line. In (B), genes or nodes coloured red are upregulated in colonised line N birds whereas those in green are upregulated in colonised line 61 birds. [file 12864_2021_7748_MOESM5_ESM.tif]

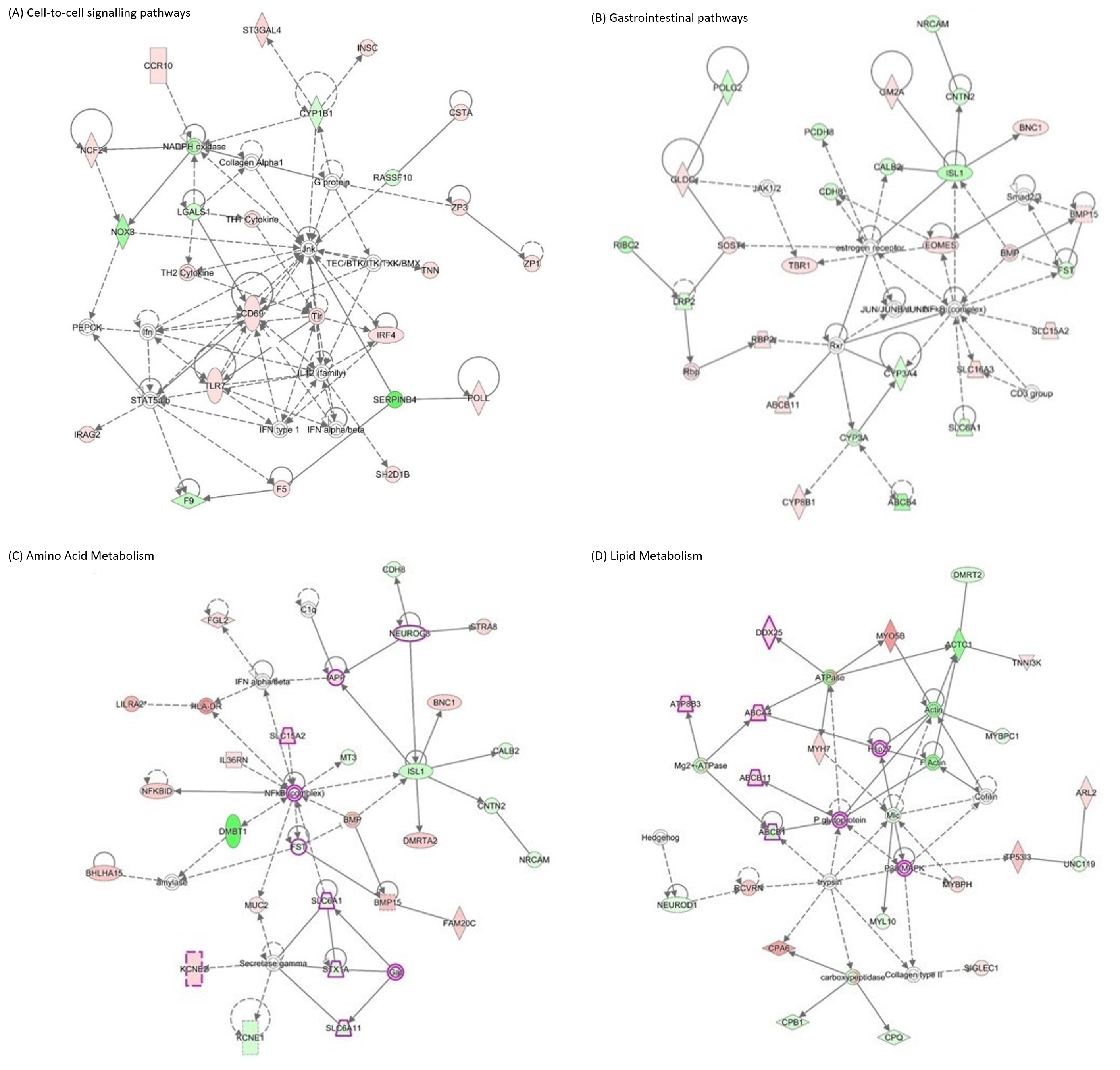

Supplement: Supplementary file 8 — Additional file 8: Figure S4. IPA of DEGs in the caecal tonsils identified between control birds of line 61 and N. Shown are significant networks of genes relating to cell-to-cell signalling (A), gastrointestinal pathways (B), amino acid metabolism (C) and lipid metabolism (D). N = 6 for each line (3 control birds pooled from each time point) Genes or nodes coloured red are upregulated in colonised line N birds whereas those in green are upregulated in colonised line 61 birds. [file 12864_2021_7748_MOESM8_ESM.tif]

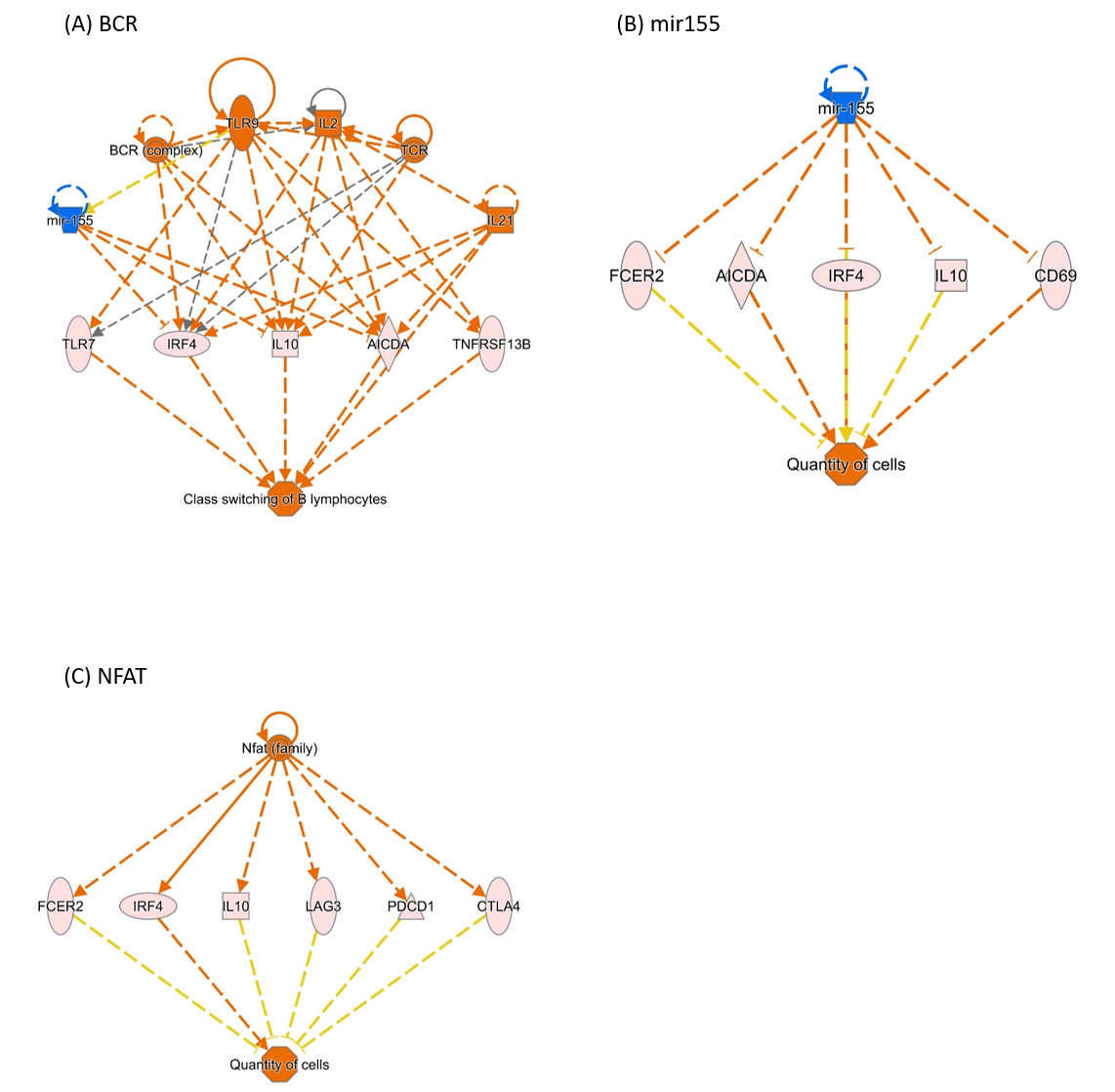

Supplement: Supplementary file 9 — Additional file 9: Figure. S5. IPA of DEGs identified between control birds of line 61 and N. Shown are significant networks associated with predicted upstream regulators of DEGs: BCR (A), mir155 (B) and NFAT (C). Genes or nodes coloured red are upregulated in line N birds whereas those in green are upregulated in line 61 birds. N = 6 for each line (3 birds pooled from each time point) [file 12864_2021_7748_MOESM9_ESM.tif]
